# Effects of platelet-rich fibrin on human endometrial stromal cells behavior in comparison to platelet-rich plasma

**DOI:** 10.3389/fcell.2024.1445928

**Published:** 2024-09-03

**Authors:** Guanghui Yuan, Duan Li, Xin Du, Yingxue Liu, Xiaoxiao Wang, Cuifang Hao

**Affiliations:** ^1^ Centre for Reproductive Medicine, Women and Children’s Hospital, Qingdao University, Qingdao, China; ^2^ Branch of Shandong Provincial Clinical Research Center for Reproductive Health, Qingdao, China; ^3^ College of Medicine, Qingdao University, Qingdao, China

**Keywords:** platelet-rich plasma, platelet-rich fibrin, endometrial stromal cells, cell apoptotic, thin endometrium

## Abstract

**Introduction:**

Intrauterine transfusion of platelet-rich plasma (PRP) has become a new treatment for thin endometrium (TE) in recent years, but its low efficacy due to rapid release of growth factors limits its clinical use. Platelet-rich fibrin (PRF) starts the coagulation cascade reaction immediately after the blood comes into contact with the test tube. The natural coagulation process results in stable platelet activation and the slow release of growth factors.

**Methods:**

In our study, primary human endometrial stromal cells (hESCs) were extracted from endometrial tissue. PRP and PRF were prepared from the patient cubital vein blood. Stromal cells were cultured in conditioned medium supplemented with PRP and PRF. Differences in cell behavior were observed by cell proliferation test and cell migration test. The relative expression levels of apoptotic Bax and antiapoptotic Bcl-2 genes were measured by qRT-PCR. The release of growth factors from PRP and PRF was detected by ELISA.

**Results:**

We found that both PRP and PRF inhibited apoptosis of hESCs, which favored cell proliferation and migration. In addition, PRF releases growth factors for a longer period of time compared to PRP.

**Discussion:**

PRF offer a more sustained therapeutic effect compared to PRP, which provides a new idea for endometrial regeneration and repair.

## 1 Introduction

The ideal endometrial thickness plays a key role in maintaining a normal pregnancy (Lessey and Young, 2019). Proliferation of the endometrial glands and angiogenesis of the uterus are the conditions of endometrial preparation before embryo implantation and are clinically reflected in the thickness of the endometrium (Casper, 2020). However, induced abortion, frequent uterine cavity surgery, and biochemical factors such as infection and hormone drugs can cause endometrial damage and endometrial regeneration disorders. They also affect implantation and survival of embryo, eventually leading to a lower pregnancy rate and increased adverse pregnancy outcome (Gameiro and Finnigan, 2017; Liu et al., 2018; Zhao et al., 2020). A thin endometrium (TE) is the main manifestation of endometrial injury (Liu et al., 2023), and its incidence rate in assisted reproductive technology (ART) is approximately 2.4% ∼ 8.5% (Kasius et al., 2014; Ranisavljevic et al., 2019). The regeneration and repair of the damaged endometrium is one of the most intractable clinical problems in the reproductive field. Current treatment modalities used for TE include medicines, such as estrogen (Carvalho MOC. et al., 2024), tamoxifen (Ji et al., 2023) and traditional Chinese medicine (Jin et al., 2023), other treatments include stem cell therapy (Wang and Tan, 2023), granulocyte colony-stimulating factor (G-CSF) (Xu et al., 2024) and intrauterine perfusion of platelet-rich plasma (PRP) (Chen et al., 2024).

PRP is a platelet concentrate obtained from autologous whole blood after centrifugation that contains a large number of growth factors, such as vascular endothelial growth factor (VEGF), transforming growth factor-β1 (TGF-β1), and platelet-derived growth factor (PDGF) (Aghajanova et al., 2018; Borhani-Haghighi and Mohamadi, 2019). PRP can promote regeneration of the injured endometrium and improve endometrial receptivity (Jang et al., 2017; Zhou et al., 2020). As a new treatment method for TE in recent years, PRP intrauterine perfusion has achieved good results (Cheng et al., 2020; Efendieva et al., 2023; Pandey et al., 2023; Pivazyan et al., 2021; Salman et al., 2023). However, its low efficacy due to rapid release of growth factors limits its clinical use. Studies have shown that the number of PRP uterine instillations correlates with the effectiveness of endometrial thickening. For those patients with endometrial thicknesses below 7 mm, multiple instillations are usually required (Pandey et al., 2023; Russell et al., 2022). It brings great physical and financial pressure to the patients. Therefore, a substitute that can slowly release growth factors is needed to treat TE.

Since Dohan et al. (2006) prepared the first PRF in 2005, it has been widely used in dentistry (Goswami et al., 2024; Özcan et al., 2021), plastic surgery (Yu et al., 2018), wound repair (Tian et al., 2023; Reksodiputro et al., 2021) and other medical fields (Amin et al., 2024; Nagaraju et al., 2017). PRF is a second-generation platelet concentrate product after PRP and is defined as an autologous leukocyte and platelet-rich fibrous biomaterial. After a single centrifugation, exogenous thrombin does not need to be added in the subsequent preparation process. In the coagulation cascade immediately initiated after blood contacts the tube, the natural coagulation process results in stable platelet activation and the slow release of growth factors. During the centrifugation of blood, fibrinogen is converted into fibrin, forming a dense three-dimensional mesh structure. This structure further protects the platelets from rapid activation, slows the release of growth factors, and can last for up to approximately 3 weeks (Lourenço et al., 2018). Previous studies have shown that PRF can promote cell migration, proliferation, differentiation, etc. (Lu et al., 2021; Wang et al., 2017). The proliferation and migration of hESCs maintain the physiological thickness of the uterus and play an important role in the window of implantation (WOI), but no studies have investigated the effect of PRF on the biological behavior of hESCs.

PRF has been used for the treatment of intrauterine adhesion (IUA) (Chen and Wang, 2022; Wang et al., 2021). What is the effect of PRF on hESCs behavior compared to PRP? In this study, we explored the release of growth factors from PRF and PRP and their effects on the behavior of hESCs. It is hoped that this study provide a new theoretical reference for the regenerative repair of endometrium.

## 2 Materials and methods

### 2.1 Volunteer recruitment

Five infertile women aged 20–40 years who underwent routine hysteroscopy before artificial assisted reproductive technology were recruited, and endometrial pathology revealed a proliferative endometrium. The exclusion criteria were as follows: 1) abnormal blood coagulation; 2) abnormal uterine structure; 3) endometrial hyperplasia; and 4) patients with severe cardiac, hepatic, pulmonary, renal, and other organ disorders. Anterior elbow vein blood was taken for the preparation of PRP and PRF, and endometrial tissue was taken for the extraction of primary hESCs. All patients voluntarily participated, were informed of the purpose and process of the experiment, and signed an informed consent form.

### 2.2 Preparation of PRP

Two 15 mL centrifuge tubes containing 1 mL of sodium citrate anticoagulant (Carvalho A. et al., 2024; Görgü et al., 2020) were prepared for each volunteer, 9 mL of venous blood was injected into each tube, and the tube was gently shaken and mixed well. After centrifugation at 300 ×g for 20 min at room temperature, the upper and lower layers of the liquid were obviously delaminated. The yellowish part of the upper layers and the junction of the upper and lower layers of the two tubes were carefully removed to form a new tube. After centrifugation at 300 ×g for another 20 min, the bottom 1 mL of liquid, namely, PRP, was removed, and the platelet concentration was adjusted to 1 × 10^9^/mL (Marx, 2001). The solution was mixed with 10% CaCL_2_ and thrombin (Sigma Aldrich, Cat: T6884) at a 9:1 volume ratio. The mixture was incubated in an incubator at 37°C for 4 h, centrifuged at room temperature at 2,000 ×g for 10 min, filtered through a 0.22 µm filter and frozen at −80°C for subsequent experiments.

### 2.3 Preparation of PRF

Of venous blood, 10 mL was placed in a special PRF glass tube without antithrombin. The test tube was immediately centrifuged at 400 ×g for 10 min (Ehrenfest et al., 2010). After rest, the blood sample can be divided into three layers. Between the red blood cell fragments in the bottom layer and the yellowish clarified liquid platelet plasma in the top layer, the yellowish gel in the middle layer is called PRF. The supernatant was discarded, and the red blood cells at the bottom of the gel were removed to obtain the primary PRF, which was then placed in a dry and sterilized container for 10 min to naturally contract and release the serum. Finally, the PRF was extruded into a film using a sterilized stainless steel box, and the PRF membrane was obtained.

The ultrastructure of the PRF scaffold was observed by scanning electron microscopy (Thermo Fisher Scientific, Apreo 2 SEM, United States). PRF specimens were fixed overnight in 2% glutaraldehyde. The next day, the sample was dried in a dryer and then sputtered with 20 nm of gold. The prepared samples were then examined under the SEM at an accelerating voltage of 10 kV to capture detailed images of the PRF scaffold microstructure.

### 2.4 Isolation, culture and identification of primary hESCs

The obtained endometrial tissue was repeatedly washed with aseptic 1 × PBS and cut into <1 mm^3^ fragments. Then added trypsin (0.25%, Sigma Aldrich, Cat: T4549) equivalent to 5 times the volume of the tissue to digest the tissue. The mixture was digested in a water bath at 37°C for 1 h (Jividen et al., 2014). The mixture was centrifuged and the tissue was resuspended with PBS. The endometrial tissue suspension was filtered through a 400 mesh filter (Fisher Scientific, Cat: NC0073140) (Lin et al., 2022) and the filtrate was collected. Re-centrifugation was performed to remove residual trypsin. The obtained cells were resuspended with DMEM medium containing 10% fetal bovine serum and seeded in a Petri dish. Primary cells were identified by immunofluorescence, and the specific marker molecule was vimentin (Vim). The cells were fixed with 4% paraformaldehyde for 15 min after being exposed to coverslips, permeabilized with 0.1% Triton X-100 for 10 min, and blocked with 5% BSA for 1 h. Subsequently, the cells were incubated with an anti-Vim antibody (1:200 dilution, Cell Signaling Technology, Cat: 5741) at 4°C overnight, followed by incubation with an Alexa Fluor 488-conjugated secondary antibody (1:500 dilution, Thermo Fisher Scientific, Cat: A11001) at room temperature for 1 h. Nuclei were stained with DAPI. Images were captured using a confocal microscope, and fluorescence intensity was analyzed using ImageJ software.

### 2.5 Preparation of conditioned medium

PRP and PRF membranes were transferred to 50 mL centrifuge tubes containing 45 mL of serum-free DMEM and incubated at 4°C for 1 day or 14 days to allow the accumulation of released growth factors. After 1 day and 14 days, the supernatant was collected by centrifugation at 1,000 rpm for 5 min and then filtered through a 0.22 μm filter. A 1% penicillin streptomycin mixture was added, and the conditioned medium was obtained, which was labeled “1dPRP/1dPRF/14dPRP/14dPRF.”

### 2.6 Cell proliferation assay

hESCs were digested with trypsin from passage 3 to generation 5 (Yang et al., 2023), counted and seeded into 4 96-well plates at 2,000 cells per well. After 24 h of incubation, the serum-free DMEM and the four conditioned media were replaced, and the cells were cultured in an incubator. One plate cell was removed at 24 h, 48 h, 72 h and 96 h. According to the instructions of the kit (Beyotime, Cat: C0038), 10 μL of CCK-8 solution was added to each well. Then place the 96-well plate in the cell incubator and incubate for 2 h. During this process, the WST-8 in the CCK-8 reagent is reduced by dehydrogenases in live cells, producing a water-soluble formazan dye. The intensity of the dye solution’s color is directly proportional to cell viability. And the OD was measured with a spectrophotometer at a wavelength of 450 nm.

### 2.7 Cell migration assay

The hESC cell suspension was seeded into the upper chamber of a 6-well Transwell plate (Costar, Cat: 3422, 8.0 μm pore size) at a density of 2 × 10^5^ cells/mL, and serum-free DMEM, and each group of conditioned media were added to the lower chamber and cultured in a CO_2_ incubator at 37°C for 24 h. The Transwell chamber was removed, the cells were rinsed with PBS, fixed with 4% paraformaldehyde solution, the unmigrated cells on top surface of the membrane were removed with a cotton swab, and the cells at the bottom surface of the membrane were rinsed with PBS and stained with 0.1% crystal violet (Justus et al., 2023). The cells were observed and imaged under a microscope.

### 2.8 Real-time PCR

A rapid extraction kit for trace sample RNA (Solarbio, Cat: R1200) was used to extract sample RNA, and a reverse transcription kit (SparkJade, Cat: AG304) was used for reverse transcription. According to the instructions of the SYBR Green qPCR kit (AGbio, Cat: AG11701), the samples were amplified by Quant StudioTM5 real-time fluorescence quantitative PCR. With the GAPDH gene as an internal reference, the relative expression of apoptotic Bax and the antiapoptotic gene Bcl-2 was detected by relative quantification. The sequences of the primers used are listed in Supplementary Table S1. The experimental data were statistically analyzed according to the 2^−ΔΔ CT^ method.

### 2.9 Release of growth factors from PRP and PRF

PRP and PRF were soaked in a 6-well plate containing 5 mL of DMEM and incubated in a cell incubator. The supernatants were collected at different time points and stored at −20°C. The levels of the growth factors VEGF (Thermo Fisher, Cat: KHG0111), TGF-β1 (Thermo Fisher, Cat: BMS249-4) and PDGF-AB (Thermo Fisher, Cat: EHPDGFAB) were detected according to the instructions of the ELISA kit. The absorbance at 450 nm was measured, and the corresponding results were calculated according to the curve.

### 2.10 Statistical analysis

All experimental data were validated by three replicates and are expressed as the mean ± standard deviation (SD) using one-way ANOVA and two-way ANOVA, which were performed with GraphPad Prism 9.0 software. P values less than or equal to 0.05 were considered to indicate statistical significance.

## 3 Results

### 3.1 Characterization of PRF

**FIGURE 1:**
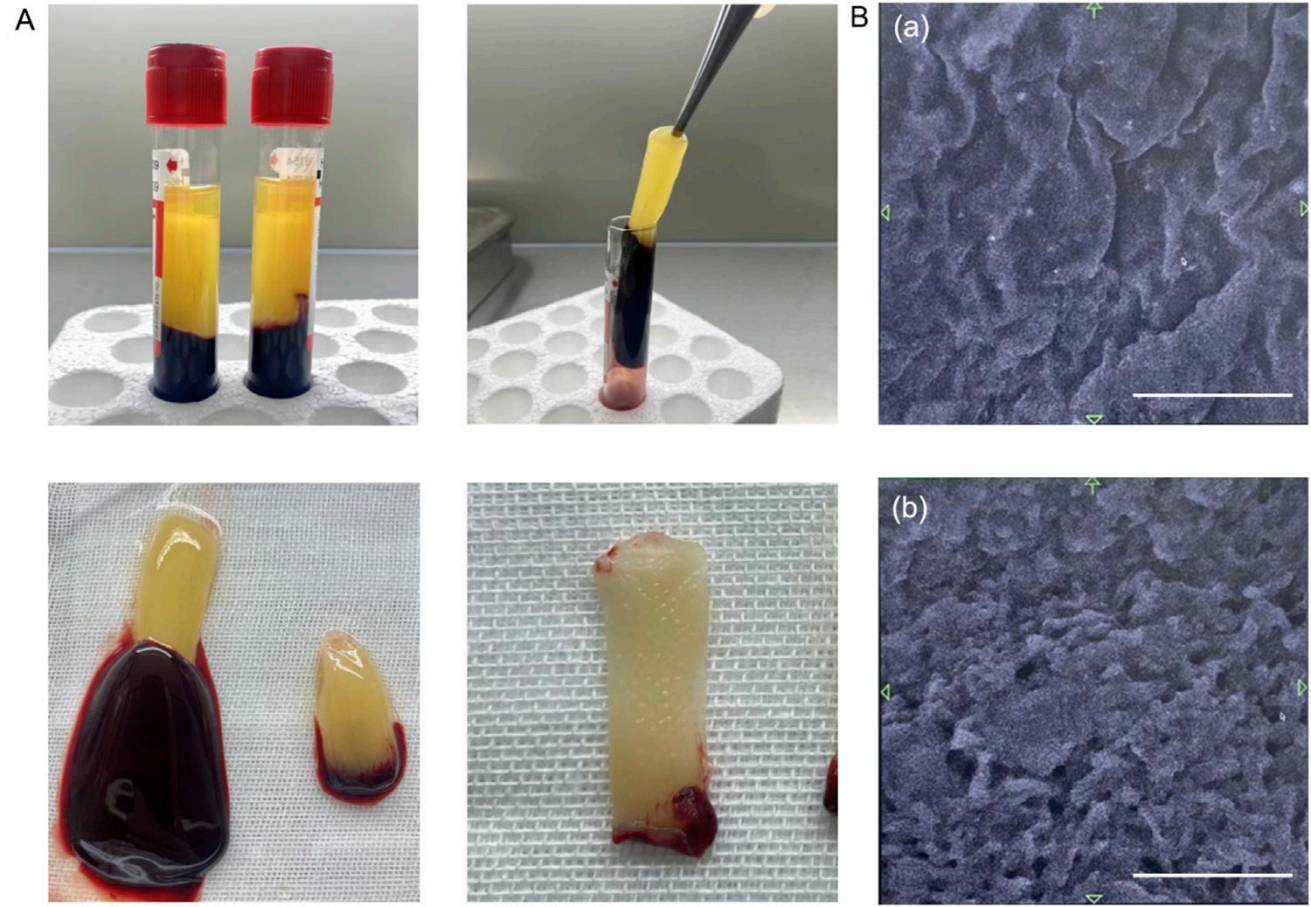
Characteristics of PRF. **(A)** PRF preparation process. **(B)** Structure of PRF under scanning electron microscope. (a) Image of the PRF surface. (b) Image of the PRF cross-section. Scale bars = 10 μm.

After centrifugation, the blood was divided into three layers: an upper colorless transparent liquid (the serum layer), a middle white jelly (the PRF layer), and a lower red clot (the erythrocyte layer). Jelly like PRF was obtained after discarding the serum and erythrocytes, and a PRF diaphragm was obtained after water removal by extrusion (Figure 1A).

Scanning electron microscopy revealed that the PRF was smooth and wrinkled overall, and further magnification revealed that it was a dense fiber network with a three-dimensional distribution. In the complex fibrin network, pores ranging from 10 μm to 30 μm are found, which are connected to each other and are expected to be useful as biological scaffolds (Figure 1B).

### 3.2 Characterization of hESCs

**FIGURE 2:**
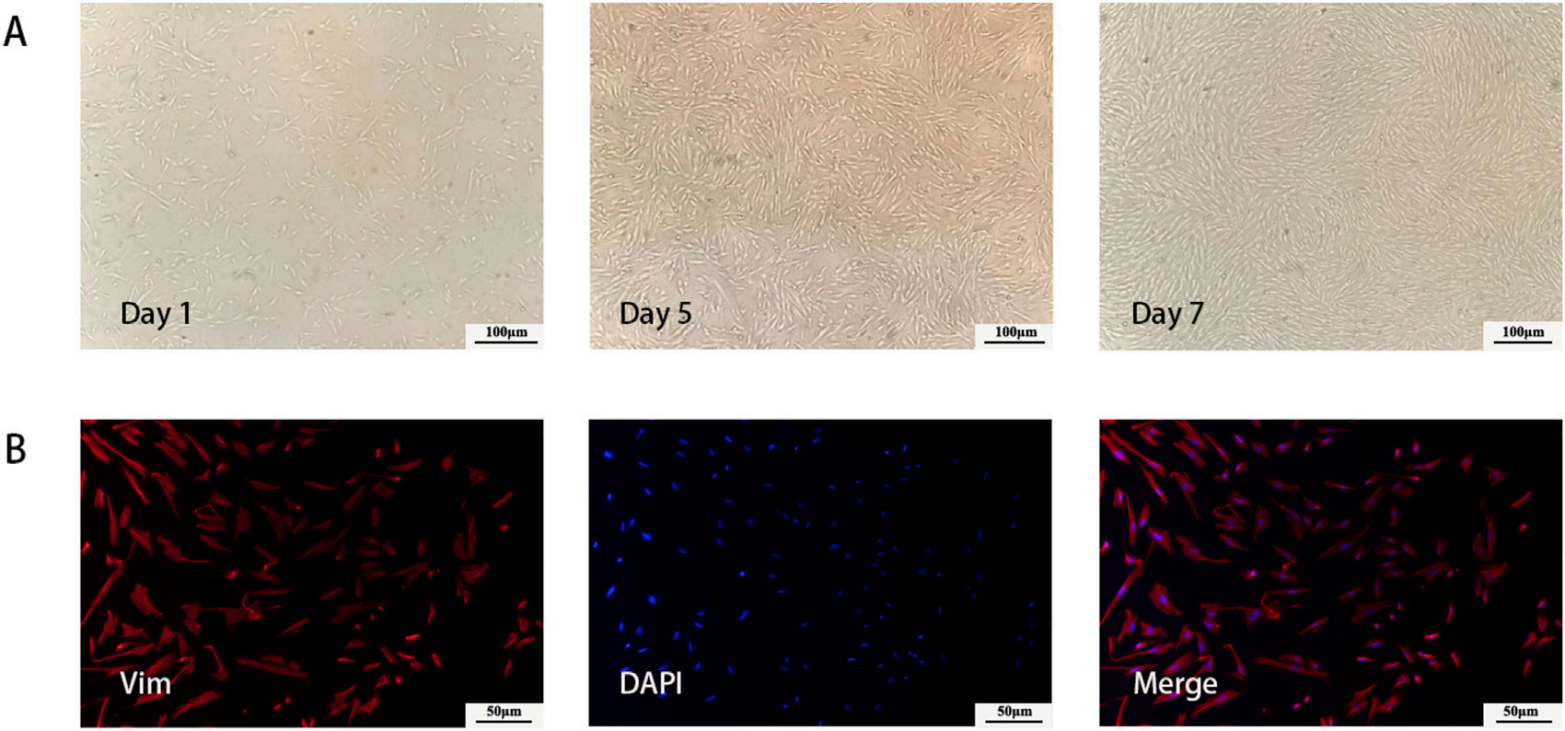
Characterization of hESCs. **(A)** Morphology of cultured primary cells at different time point. **(B)** Immunofluorescent imaging data was presented as vimentin expression after culture.

Two days after seeding, the cells had successfully adhered to the wall, and the primary cells were long and fusiform. After 4–7 days of culture, the cells grew exuberantly, closely arranged, and swirled (Figure 2A). Immunofluorescence staining revealed that vim-positive cells accounted for 95% of the total cells, confirming that the isolated cells were hESCs (Figure 2B).

### 3.3 Proliferative effects

**FIGURE 3:**
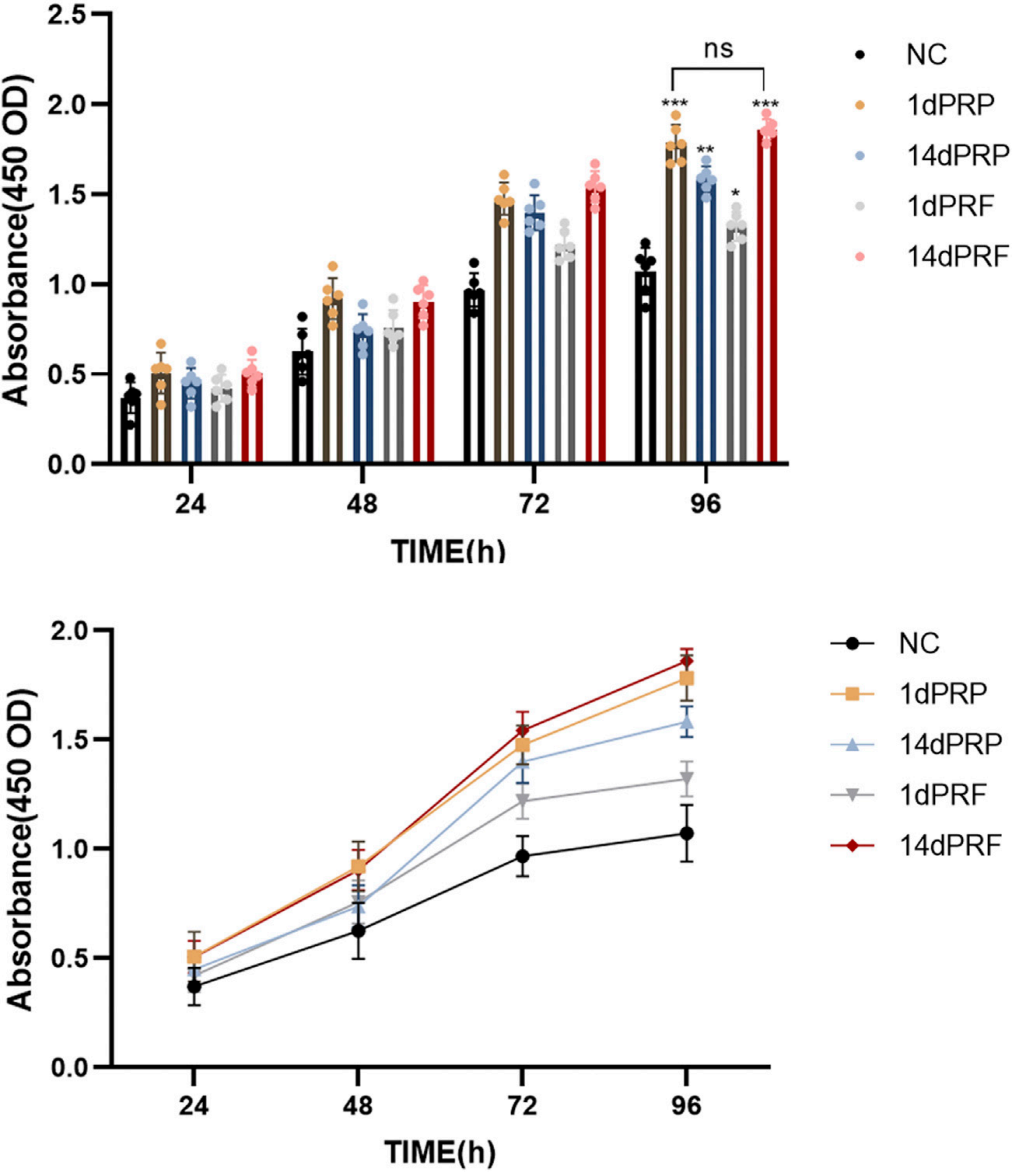
Effects of PRP and PRF on the proliferation of hESCs at 24, 48, 72 and 96 h ***P < 0.001 compared with control group; **P < 0.01 compared with control group; *P < 0.05 compared with control group.

After incubation with the four conditioned media, there was no obvious morphological change in the hESCs. As shown in Figure 3, compared with those in the control group, the four conditioned media promoted the proliferation of hESCs. The degree of proliferation of the 1dPRP- and 14dPRF-treated cells was significantly greater than that of the other conditioned medium groups. The degree of proliferation in the 14dPRF group was greater than that in the 1dPRP group, but the difference was not statistically significant.

### 3.4 Migration effects

**FIGURE 4:**
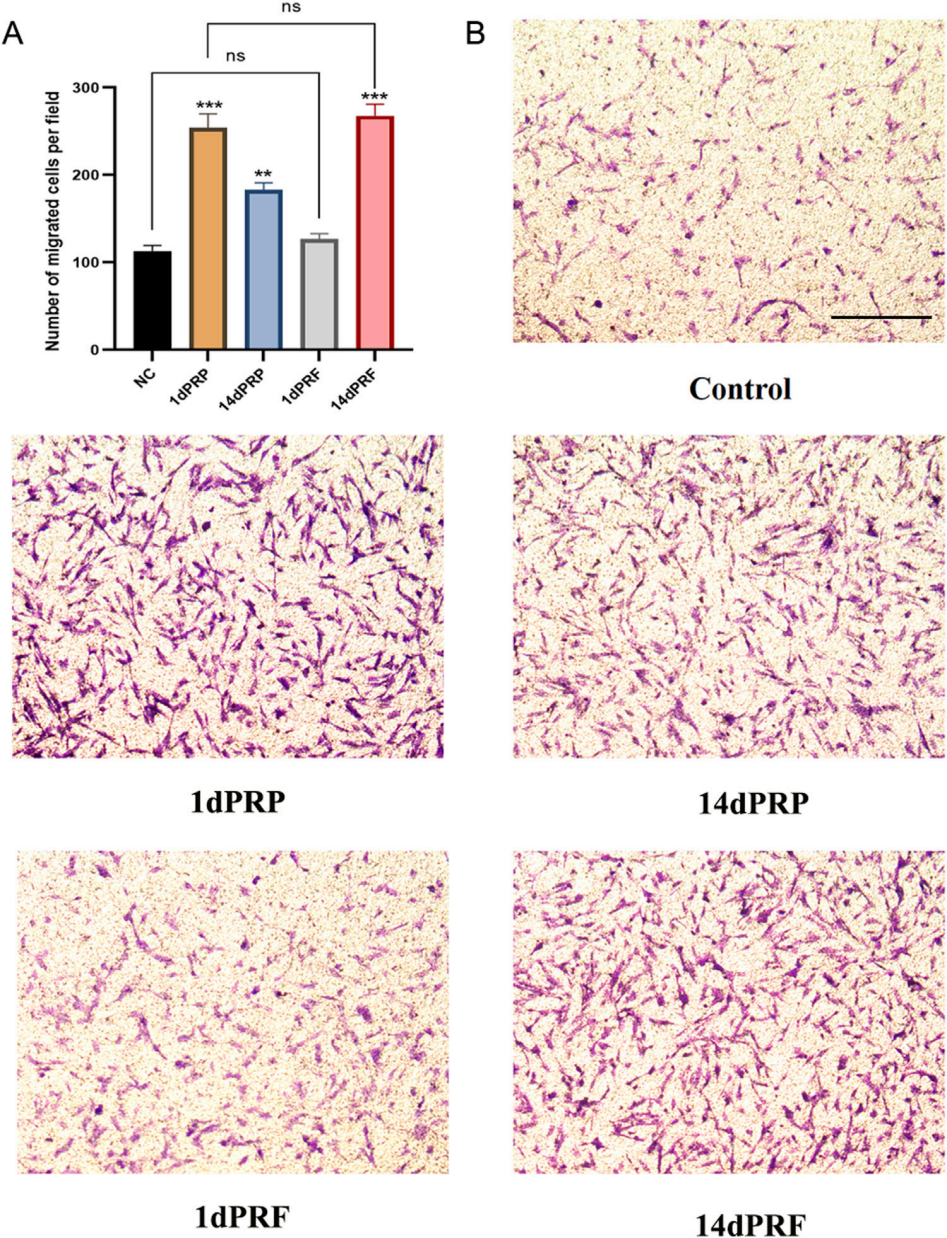
Effect of PRP and PRF on the migration of hESCs. **(A)** Normalized cell migration demonstrated a significant increase in 1dPRP and 14dPRF groups. **(B)** Cell migration was assessed after 24 h. Scale bars = 100 μm ***P < 0.001 compared with control group; **P < 0.01 compared with control group; ns, no significance.

As shown in Figure 4, all four conditioned media could stimulate hESC migration. There was no significant difference between the 1dPRF group and the control group, but there was a significant difference among the other three groups. Among these groups, the 14dPRF group showed the greatest effect on hESC migration, followed by the 1dPRP group.

### 3.5 Gene expression

**FIGURE 5:**
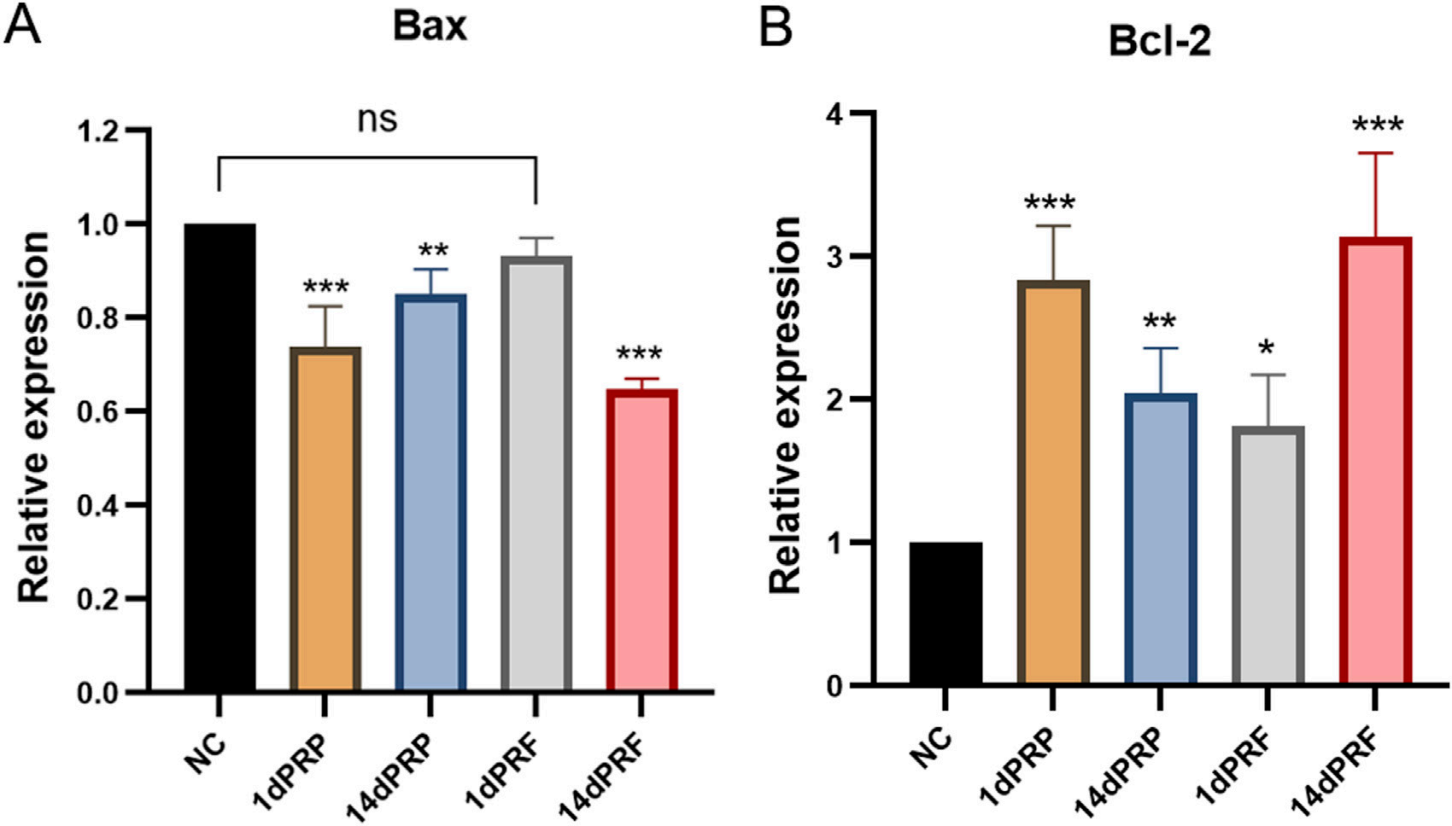
Effects of PRP and PRF on the gene expression in hESCs. **(A)** Apoptosis gene Bax expression. **(B)** Anti-apoptosis gene Bcl-2 expression. ***P < 0.001 compared with control group; **P < 0.01 compared with control group; ns: no significance.

The expression of apoptosis-related genes was measured by qRT-PCR. Compared with those in the control group, the expression of the apoptosis gene Bax was significantly decreased, and the expression of the antiapoptotic gene Bcl-2 was significantly increased in hESCs cultured in PRP- and PRF-conditioned media (*P* < 0.01). The effect of 14dPRF was the same as that of 1dPRP and was better than that of the other conditioned media (*P* < 0.001) (Figure 5).

### 3.6 Growth factor release curve

**FIGURE 6:**
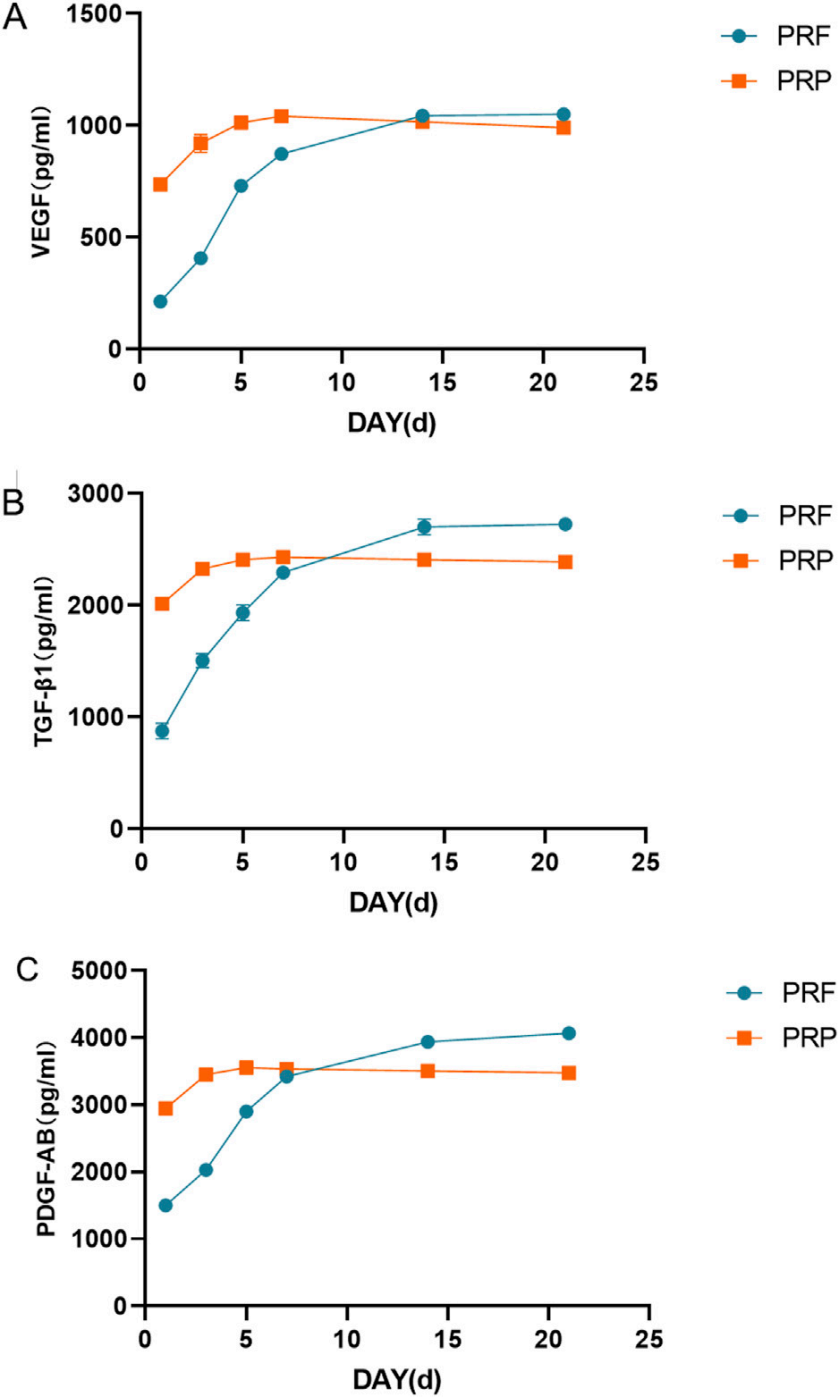
Growth factor release curve. **(A)** Total amount of VEGF released at different time points. **(B)** Total amount of TGF- β1 released at different time points. **(C)** Total amount of PDGF-AB released at different time points.

As shown in Figure 6, the initial dose of the three growth factors in PRP is relatively high, and the release is mainly concentrated in the first 3 days, which tends to be flat, and there are signs of dose reduction after 1 week. The initial amount of PRF growth factor was low, and the total amount of VEGF and TGF-β1 tended to be stable after continuous release for 2 weeks. However, PDGF-AB were still released continuously after 2 weeks. The total amount of TGF-β1 and PDGF-AB released from PRF exceeded that of PRP after 2 weeks, while the release of VEGF was about the same as that of PRP at the 3rd week.

## 4 Discussion

There has been much research into treatments for thin endometrium, each with its own advantages and disadvantages. Medication is often the first-line treatment but may cause side effects such as headaches, nausea, and breast tenderness, and long-term use can lead to drug resistance (Carvalho MOC. et al., 2024). Stem cell therapy shows promise for regenerating the endometrium but is still experimental (Wang and Tan, 2023). G-CSF intrauterine perfusion is newer approaches that aim to enhance endometrial growth, with limited long-term data (Banerjee et al., 2022). PRP intrauterine perfusion has achieved good results in recent years, but multiple instillations are usually required in clinical application (Chen et al., 2024; Pandey et al., 2023; Russell et al., 2022). Considering the disadvantage that PRP growth factors cannot be released for a long period of time, PRF turns out to be an alternative worth investigating.

With the emergence of second-generation PRF, researchers have found that compared with traditional PRP, PRF has many advantages (Miron et al., 2017). First, PRF is easy to prepare. Its preparation is simplified to one-step centrifugation, which requires centrifugation at a low speed, simulates the physiological coagulation process and allows the collection of natural clots (Lundquist et al., 2018). Second, PRF does not use any exogenous additives in the preparation process, which avoids the risk of immune rejection caused by thrombin used in PRP preparation. This also avoids the spread of infectious diseases and coagulation dysfunction (Anitua et al., 2016). Finally, from an ultrastructural point of view, the maturity of the fibrin reticular structure of PRF is better than that of PRP, which is related to the release of the growth factors. Specifically, PRP is in a liquid state, leading to a faster release rate of growth factors and consequently a shorter duration of action. In contrast, PRF has a three-dimensional fibrin structure that provides a stable scaffold, acting as a reservoir for various growth factors. This structure allows for the prolonged release of growth factors, ensuring their sustained activity over a more extended period (Kobayashi et al., 2016; Zwittnig et al., 2022).

Considering the above advantages of PRF, we explored its effects on hESCs. In this study, the conditioned medium extracts at various time points demonstrated that the medium obtained after 14 days of PRF immersion significantly promoted the proliferation and migration of hESCs. Several studies have shown that PRF can significantly promote the proliferation of various cell types, including bone marrow mesenchymal stem cells (Sirous et al., 2024), skin fibroblasts (Li et al., 2022), and gingival fibroblasts (Pitzurra et al., 2020). These cells exhibit increased proliferation and migration rates under stimulation with PRF. In comparison, although PRP can also promote cell proliferation, its effects are generally less pronounced than those of PRF (Masuki et al., 2016). Currently, there are no comparative studies on the effects of PRF and PRP on human endometrial stromal cells.

Currently there are many studies on the mechanism of action of PRP in promoting endometrial regeneration. PRP can promote cell mitosis by activating the signal pathway on the surface of the cell membrane, resulting in the proliferation of endometrial functional layer epithelial cells, the increase of glandular epithelium and the proliferation and expansion of spiral arteries, thus promoting the growth of endometrial cells (Mao et al., 2023). It can also inhibit the release of inflammatory factors in injured endometrium and mediate the expression of interleukin-10, thus favoring the regeneration of the endometrium, restorating of homeostatic conditions after endometrium injury (Choi et al., 2023; Jang et al., 2017; Zhou et al., 2020). Because the composition of PRF is similar to PRP, in addition to its anti-inflammatory effects, PRF also can regulate intercellular signaling through the release of growth factors. Through the activation and regulation of signaling pathways such as Wnt/β-catenin (Zhang et al., 2016), JAK/STAT (Fitzgerald et al., 2020) and Notch (Huang and Ogawa, 2019), PRF can promote cell activity and function. Superior to PRP, the fibrin and leukocytes contained in the PRF also provide additional support and immunomodulatory functions (Narayanaswamy et al., 2023). This study found that expression of apoptotic gene Bax decreased and expression of anti-apoptotic gene Bcl-2 increased significantly, suggesting that PRF and PRP can effectively inhibit the apoptosis of hESCs. Inhibition of apoptosis can maintain cell viability and provide more opportunities for cell proliferation and migration (Hong et al., 2023). In addition, reducing apoptosis can promote cell migration by improving the environment of the extracellular matrix (He et al., 2023).

The concentrations of three growth factors released by PRF and PRP at different time points were detected, and it was found that the total amount of VEGF and TGF- β1 tended to be stable after continuous release for 2 weeks. However, PDGF-AB were still released continuously after 2 weeks. The total amount of TGF- β1 and PDGF-AB released from PRF was higher than that of PRP after 2 weeks. VEGF, TGF-β1 and PDGF play a key role in the functional regulation of hESCs. VEGF stimulates the cells to secrete more angiogenic factors and enhances cell-cell interactions, thus promoting the formation of neovascularization. It also activates the cell proliferation signaling pathway and promotes proliferation and migration (Abhinand et al., 2016). TGF-β1 regulates cellular immune function, reduces inflammatory responses, and maintains the stability of the intrauterine environment. It also stimulates the synthesis and secretion of extracellular matrix components, such as collagen and fibronectin, which are essential for the maintenance of the structure and function of the endometrium (Mantel and Schmidt-Weber, 2011; Yang et al., 2021). PDGF activates downstream signaling pathways through its receptors to enhance cell proliferation and migration. It also plays an important role in regulating extracellular matrix remodeling (Wang et al., 2023). The current related studies have large differences in the results of PRP and PRF growth factor release (Kobayashi et al., 2016; Kushida et al., 2014; Mochizuki et al., 2022). This may be related to individual differences in samples, preparation methods, and experimental conditions. The present study examined the concentration of growth factors within the supernatant and focused on the comparison of PRP and PRF release duration. In the present study, we found that PRF can release these growth factors in a long-lasting manner, which is beneficial to cell proliferation and migration.

In summary, the release of growth factors from PRF is longer than that from PRP, and PRF without exogenous anticoagulant can affect the behavior of hESCs more significantly by affecting the proliferation, migration, and more inhibition of apoptosis of hESCs. Compared with PRP, PRF can significantly prolong endometrial tissue repair. However, this study has several limitations. First, only the effects of PRF on cells were investigated, necessitating further animal and clinical trials to confirm the safety and efficacy of PRF. Second, the study used normal endometrial cells, and future research should focus on endometrial stromal cells from patients with TE for more relevant insights. Finally, we did not explore all critical markers and pathways affecting the impact of PRF on endometrial stromal cells. Future studies will address these gaps and will be pivotal in determining the feasibility and effectiveness of PRF as a therapeutic alternative.

## Data Availability

The raw data supporting the conclusions of this article will be made available by the authors, without undue reservation.
