# Supplementary material for: Effects of platelet-rich fibrin on human endometrial stromal cells behavior in comparison to platelet-rich plasma
Source: Front Cell Dev Biol. 2024 Sep 3;12:1445928. doi: 10.3389/fcell.2024.1445928 (PMC11405248; doi:10.3389/fcell.2024.1445928)
Supplement: Supplementary file 1 [file Table1.DOCX]

Supplementary Material

**Supplementary Table 1.** PrimerSeq

| Gene | upstream | downstream |
| --- | --- | --- |
| Bax | 5’-CCCGAGAGGTCTTTTTCCGAG-3’ | 5’-CCAGCCCATGATGGTTCTGAT-3’ |
| Bcl-2 | 5’-CATGTGTGTGGAGAGCGTCA-3’ | 5’-CACTTGTGGCTCAGATAGGCA-3’ |
| GAPDH | 5’-AGAAGGCTGGGGCTCATTTG-3’ | 5’-AGGGGCCATCCACAGTCTTC-3’ |
